# Metrics of arterial hyperoxia and associated outcome in critical care

**DOI:** 10.1186/2197-425X-3-S1-A661

**Published:** 2015-10-01

**Authors:** HJF Helmerhorst, DL Arts, E de Jonge, DJ van Westerloo

**Affiliations:** Leiden University Medical Center, Department of Intensive Care Medicine, Leiden, Netherlands; Academic Medical Center, Department of Medical Informatics, Amsterdam, Netherlands

## Introduction

In recent years, emerging evidence has shown the potential risks of arterial hyperoxia, but observational studies failed to indisputably demonstrate its impact on clinical outcome of critically ill patients. Importantly, the lack of a clinical definition of hyperoxia and methodological limitations hamper the interpretation and clinical relevance of these studies.

## Objectives

We aimed to systematically evaluate previously used metrics for defining hyperoxia and associations with outcome of intensive care unit (ICU) admission.

## Methods

Arterial blood gas (ABG) analyses between July 2011 and July 2014 were extracted from the patient data management system (PDMS) database of three large tertiary care ICUs in the Netherlands. Data from all admissions where more than one ABG was available were supplemented with anonymous demographic and admission and discharge data from the Dutch National Intensive Care Evaluation (NICE) registry. Identified oxygen metrics from a systematic review of the literature included the first, highest, worst, and mean arterial oxygen tension (PaO_2_). Worst PaO_2_ was defined as the PaO_2_ associated with the lowest PaO_2_/FiO_2_ ratio. In logistic regression models, we analysed the associations between hospital mortality and severe hyperoxia (PaO_2_> 200 mmHg) defined by either identified metric, calculated over the total ICU admission.

## Results

Overall, 14,464 patients were included of whom 1,991 (13.7%) died before hospital discharge. All identified metrics showed statistically significant associations between severe hyperoxia and hospital mortality. The risk estimates differed substantially between the metrics used for defining hyperoxia (P < 0.001). The strongest effects were found for the mean and the highest PaO_2_: crude odds ratio 3.20 [95% CI 2.21-4.58] and 2.81 [95% CI 2.49-3.18], respectively. The effect was weaker for the first and worst PaO_2_: crude odds ratio 1.22 [95% CI 1.05-1.41] and 1.17 [95% CI 1.00-1.36], respectively.

## Conclusions

In this multicenter cohort study, severe arterial hyperoxia was associated with hospital mortality and this association was independent of the used metric for hyperoxia. However, the metric choice greatly influences the effect size for hospital outcome.

## Grant Acknowledgment

This work was financially supported by an unrestricted grant issued by the Netherlands Organization for Health Research and Development (ZonMw).

ADDIN EN.REFLIST

